# Moderation Effect of Emotional Expressivity on the Associations Between Schizotypal Traits, Autistic Traits and Social Pleasure

**DOI:** 10.1002/pchj.70003

**Published:** 2025-02-24

**Authors:** Li‐Ying Zhang, Miao Wang, Xin‐Wei Fu, Shou‐Nuo Chen, Jie Gu, Shuai‐Biao Li, Min‐Yi Chu, Yan‐Yu Wang, Yi Wang, Raymond C. K. Chan

**Affiliations:** ^1^ School of Psychology Shandong Second Medical University Weifang China; ^2^ Neuropsychology and Applied Cognitive Neuroscience Laboratory, CAS Key Laboratory of Mental Health Institute of Psychology, Chinese Academy of Sciences Beijing China; ^3^ Department of Psychology University of Chinese Academy of Sciences Beijing China; ^4^ Shanghai Mental Health Center Shanghai Jiao Tong University School of Medicine Shanghai China

**Keywords:** angry, autistic trait, facial expressions, schizotypy, social pleasure

## Abstract

Diminished social pleasure has been reported in people with schizophrenia and autism spectrum disorder (ASD). Previous studies suggested that emotional expressivity is closely correlated with social pleasure. However, the underlying psychological mechanisms between traits related to schizophrenia and ASD, emotional expressivity, and social pleasure remain unclear. This study aimed to investigate the relationship between subclinical schizotypal and autistic traits, facial expressions, and social pleasure. Eighty‐six healthy participants (mean age = 20.35 ± 0.26 years, 44 males) were recruited to complete an emotion elicitation task and an autobiographical recalling task, while their facial expressions were videotaped for computerized analysis using the FaceReader. The intensity of different facial expressions (happy, sad, angry, surprised, scared, and disgusted), valence, and arousal were extracted. The self‐report Multidimensional Schizotypy Scale (MSS), Autism‐Spectrum Quotient (AQ), and Anticipatory and Consummatory Interpersonal Pleasure Scale (ACIPS) were administered to measure subclinical traits and social pleasure. Partial correlation analysis and moderation analysis were performed. Both schizotypal and autistic traits were negatively correlated with social pleasure. The moderation effects of angry facial expression for both schizotypal and autistic traits on their associations with social pleasure were significant. In addition, scared and surprised facial expressions moderated the associations between positive and negative dimensions of schizotypy and social pleasure, while arousal moderated the associations between autistic traits and social pleasure. Our study identified different moderating effects of facial emotion expressions on schizotypal and social anhedonia and autistic traits and social anhedonia, thereby revealing possible different psychopathological mechanisms underlying similar social anhedonia in subclinical populations.

## 1 Introduction

Impaired social functioning is common in patients with schizophrenia (Green et al. 2024; Mow et al. 2020) and autism spectrum disorder (ASD) (Banker et al. 2021; Mikami et al. 2019; Pelphrey et al. 2011). Diminished social pleasure has been reported to contribute to poor social functioning in the two disorders (Abel and Minor 2023; Feller et al. 2021; Han et al. 2019). Social Pleasure refers to pleasant experiences from social interactions (Berridge and Kringelbach 2015; Gooding and Pflum 2014). Empirical studies and meta‐analyses have consistently reported reduced social pleasure in patients with schizophrenia (Ritsner et al. 2018; Liang et al. 2020; Wang et al. 2014; Abel et al. 2023; Abel and Minor 2023) and ASD (Gadow and Garman 2020; Han et al. 2019). Diminished social pleasure and impaired social functioning may be transdiagnostic features across schizophrenia and ASD (Barkus and Badcock 2019; Jutla et al. 2022). Subclinical studies that focused on people with schizotypal traits or autistic traits can minimize potential confounding factors such as medication and duration of illness in clinical cases (Barrantes‐Vidal et al. 2015; Bralten et al. 2018). Recent findings suggested that people with a high level of schizotypal traits (Chan et al. 2012; Gooding et al. 2014; Wang et al. 2014) and autistic traits (Novacek et al. 2016) also exhibited diminished social pleasure. Network analysis showed that both schizotypal and autistic traits were closely related to reduced social pleasure (Zhang et al. 2020). However, few studies have adopted a transdiagnostic approach to examine psychological mechanisms underlying impaired social pleasure or social anhedonia in people with high levels of schizotypal and autistic traits.

Emotion expression involves observable clues (including facial emotion, vocal, verbal, and symbolic expressions) that indicate the presence of internal emotional state of self or others (van Kleef and Côté 2022). According to the “Emotions as Social Information” (EASI) theory, an individual's emotion expressions do not only show his/her internal emotional experiences, but also display social motives; and both facets together could influence other's feelings, thoughts, and behaviors (Van Kleef 2009). A meta‐analysis indicated that emotion expressions significantly influenced social and interpersonal outcomes, with expression of positive and non‐specific emotion associated with better social outcomes, whereas expression of negative emotion (angry in particular) was related to poor social outcomes (Chervonsky and Hunt 2017). Moreover, previous research showed that both positive and negative expressivity of emotion were associated with higher level of social pleasure (Gooding et al. 2015). Compared to healthy controls, people with high level of social anhedonia reported lower scores on positive and negative expressivity of emotion, and displayed fewer numbers of positive facial expressions per minute in response to film clips (Leung et al. 2010).

Given the impaired emotion expressions in both schizophrenia (Mandal et al. 2023) and ASD patients (Trevisan et al. 2018), and the close relationship between emotion expression and social pleasure, we proposed that abnormal emotion expressions may be moderating influencers contributing to diminished social pleasure. Abnormal emotional expressivity has been regarded as negative symptoms in schizophrenia, according to second‐generation negative symptom scales, such as the Clinical Assessment Interview for Negative Symptoms (CAINS) (Horan et al. 2011; Chan et al. 2015) and the Brief Negative Symptom Scale (BNSS) (Kirkpatrick et al. 2011). Patients with schizophrenia showed less frequent positive and more frequent negative facial expressions when watching positive and negative film clips, respectively (Sestito et al. 2013). Moreover, schizophrenia patients displayed less frequent smiles during “autobiographical recalling” of positive and negative events (Trémeau et al. 2005). Regarding ASD, previous laboratory studies found that ASD patients showed lower intensity of positive facial expression when watching positive film clips (Bangerter et al. 2020), yet higher intensity of happy, angry, and sad expressions, and lower intensity of scared expressions during “autobiographical recalling” relative to controls (Faso et al. 2015). A previous review indicated that people with high schizotypal traits showed attenuated emotional expressions both in self‐report and prosodic expressivity (Giakoumaki 2016) and exhibited diminished acoustic expression to neutral pictures compared to healthy controls (Cohen et al. 2009). On the other hand, people with high autistic traits mimicked happy faces more, under no joint attention social conditions, compared to people with low autistic traits (Neufeld et al. 2016). Such findings suggested that autistic traits were associated with poor emotion expression under social conditions.

Recently, automated facial analysis software has been developed using the Facial Action Coding System (FACS) (Ekman and Friesen 1976) and machine learning algorithms to recognize facial expressions. Compared to manual coding by human raters, automated facial analysis software like FaceReader offers comparable or even superior accuracy for basic emotions and is not subjected to raters' bias and fatigue (Skiendziel et al. 2019). Several previous studies applied automated facial analysis to patients with schizophrenia and ASD and showed interesting results. For example, schizophrenia patients presented less negative and positive facial expressions when recalling emotional experiences during the past hour in daily life (Cohen et al. 2020). Compared with controls, children with ASD showed less intensity of happy, sad, and scared facial expressions in the mimicry task (Liu et al. 2023) and exhibited less intensity of surprised, scared, angry, and happy expressions when watching social emotional scenes (Turan et al. 2023).

Taken together, few studies have examined the associations among facial emotion expressions, social pleasure, and subclinical traits (i.e., schizotypal and autistic traits). The current study aimed to investigate the associations between schizotypal and autistic traits with social pleasure and to explore the respective relationships between schizotypal and autistic traits with computerized analysis of facial emotion expressions during film‐clip watching and autobiographical recalling tasks. It also aimed to examine the moderation effect of facial expressions on the relationships between subclinical traits and social pleasure to explore the potential psychological mechanisms. Based on the existing findings in schizophrenia (Wang et al. 2014; Sestito et al. 2013; Cohen et al. 2020) and ASD (Novacek et al. 2016; Faso et al. 2015; Bangerter et al. 2020; Turan et al. 2023), we hypothesized that (1) both schizotypal and autistic traits would be negatively correlated with social pleasure; (2) schizotypal traits would be inversely correlated with the intensity of positive‐valenced facial expressions but positively correlated with the intensity of negative‐valenced facial expressions when watching film‐clips or during autobiographical recalling tasks; and (3) autistic traits would be inversely correlated with both positive‐ and negative‐valenced facial expressions during film watching but positively correlated with both positive‐ and negative‐valenced facial expressions (such as angry, sad) during autobiographical recalling.

## 2 Methods

### 2.1 Participants

Given that estimates of the moderation effect of facial expressions on the association between schizotypal/autistic traits and social pleasure were lacking in the extant literature, the required sample size was estimated according to the reported effect sizes of correlation analysis between schizotypal/autistic traits and social pleasure (DeBats et al. 2024; Novacek et al. 2016). Using the G*Power software (Faul et al. 2007), we found the minimum sample size for correlation analysis with schizotypal traits to be 74 participants (with *ρ* = 0.34, *α* = 0. 05, and 85% power) and that for correlation analysis with autistic traits to be 39 participants (with *ρ* = 0.46, *α* = 0. 05, and 85% power). In this study, we recruited 86 participants (42 males, mean age = 20.35 ± 0.26, age range 17–23) from Shandong Second Medical University. All participants were right‐handed and did not have any current or past history of psychiatric disorder, based on the Mini‐international Neuropsychiatric Interview (MINI) (Lecrubier et al. 1997; Si et al. 2009). This study was approved by the ethics committee of Shandong Second Medical University (No. 2023YX080). Written informed consent had been obtained before the study began.

### 2.2 Measures

#### 2.2.1 Video Recording and Computerized Analysis for Facial Expressions

Two different tasks for facial expression recording were used, that is, (1) emotion elicitation by film clips, and (2) autobiographical recalling of emotional events. In the *emotion elicitation task*, participants were instructed to watch six movie clips played on the screen of a computer. We selected two movie clips for neutral valence (clip 1 = 2.94; clip 2 = 3.43 [1 = *very negative*, 9 = *very positive*]) and arousal (clip 1 = 3.00, clip 2 = 3.51 [1 = *very low*, 9 = *very high*]), two movie clips for negative valence (clip 3 = 1.26; clip 4 = 2.26) and arousal (clip 3 = 7.24; clip 4 = 6.06), and two movie clips for positive valence (clip 5 = 7.03; clip 6 = 7.71) and arousal (clip 5 = 7.09; clip 6 = 7.37) from a well‐established Chinese emotional film database (Ge et al. 2019). The duration of each move clip lasted for 32–113 s. Participants first watched a neutral video, followed by two negative videos, then watched another neutral video followed by two positive videos. The order of the two negative videos was counterbalanced among participants, the same for the two positive videos. After watching each movie clip, participants completed a 9‐point Likert scale (1 = *very negative*, 9 = *very positive*) to assess their subjective emotional valence and arousal in response to the stimuli. In our study, the self‐reported valence and arousal ratings for each movie clip were listed as follows: valence and arousal of Neutral Clip 1 was 4.85 and 0.89, whereas valence and arousal for Neutral Clip 2 was 4.95 and 0.58. Valence and arousal of Negative Clip 3 was 2.65 and 1.38, whereas valence and arousal of Negative Clip 4 was 3.94 and 1.04. Valence and arousal of Positive Clip 5 was 6.53 and 1.25, whereas valence and arousal of Positive Clip 6 was 7.00 and 1.25.

In the *autobiographical recalling task*, participants were instructed to narrate 2 positive‐ and 2 negative‐valenced events, each lasting for less than 3 min. Using the mood‐induction method (Mills and D'Mello 2014), participants were instructed to recall their most pleasant and least pleasant experiences, and then immerse themselves in the scenario while they were narrating. During video playing or autobiographical recalling phases of both tasks, a camera was set to record participants' facial expressions (above the shoulders), with a 1920 × 1080 HD resolution, at a distance of 50 cm The recording was conducted under natural daylight, and participants should not cover their faces. To ensure participants felt positive upon completion of the experiment, they completed film elicitation and autobiographical recalling for negative emotions first, followed by the two phases for positive emotions with 10 min in between. Participants were asked to report their emotions by completing the PANAS scale at different specified time points, that is, (1) immediately before the experiments, (2) having completed the negative emotional tasks, and (3) having finished the positive emotional tasks. In total, we collected 6 video recordings of the film elicitation task and 4 recordings of the autobiographical recalling task for each participant.

**FIGURE 1:**
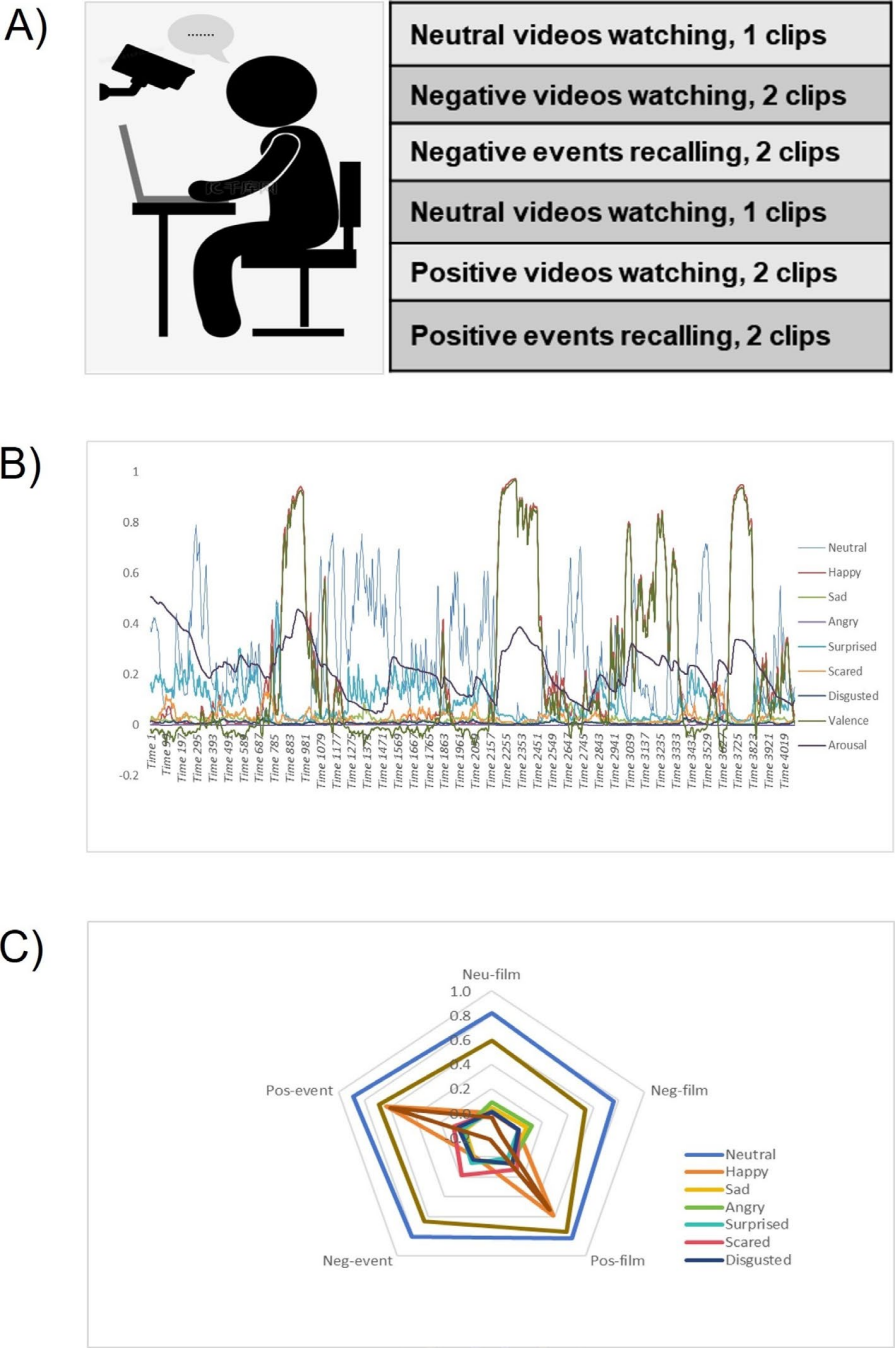
The input and output for computerized analysis of facial expression. Flow diagram of Recording and analysis of facial expressions under different conditions (taking the data of one participant as an example). (A) Facial expression recording under five conditions, number of video clips of recoding; (B) The specific facial expressions, valence, and arousal values under a positive event recalling condition analyzed by FaceReader v6.0, taking one participant as an example; (C) Calculating average of highest 10% of the intensity for specific facial expressions, and arousal; highest (or lowest) 10% of valence during positive and neutral emotion (or negative) under five conditions (Neu/Neg/Pos‐film: Neutral/Negative/Positive film watching; Neg/Pos‐event: Negative/Positive event recalling).

The FaceReader version 6 (Noldus 2014) was adopted to analyze facial expressions in video recordings. The FaceReader captured faces through various Action Units (AUS), and synthesized an artificial face model using the Active Appearance Model. The face model was classified into different categories. The neural network algorithm demonstrated an accuracy of 89% (Den Uyl and Van Kuilenburg 2005) and good validation (Lewinski et al. 2014a). The FaceReader analyzed videos at each frame (sample rate of 30 Hz) using deep learning algorithms, and generated output of the intensity of specific facial expressions, the valence and arousal of facial expressions, and other variables (e.g., indicating whether the eyes were open or closed, the eyebrows were raised, neutral or lowered). In our study, we used the data that provided by the FaceReader regarding (1) emotional intensity (ranged 0–1, with 0 = *not at all*, 1 = *maximum*) for facial emotion expressions, including happy, sad, angry, surprised, scared, disgusted, and neutral expressions, (2) emotional valence (1 = *extremely negative*, 9 = *extremely positive*), and (3) emotional arousal (0 = *not active*, 1 = *maximally active*). If facial expressions in more than 20% of the frames could not be recognized, the data of the corresponding video recording would be excluded. Following the previous study (Landmann 2023; Lewinski et al. 2014b), we calculated the average intensity of top 10% data for each specific facial expression (happy, sad, angry, surprised, scared, and disgusted and neutral) along with arousal and valence. Notably, we used averaged valence of bottom 10% data during negative emotion elicitation by films and autobiographical recalling conditions. Figure 1 shows the input and output for computerized analysis of facial expressions using the FaceReader v6.

#### 2.2.2 Self‐Report Scales

The Multidimensional Schizotypy Scale (MSS) (Kwapil et al. 2018) is a 77‐item self‐report scale for assessing the positive, negative, and disorganized dimensions of schizotypal traits. The MSS has good reliability and validity in a Chinese sample (Wang et al. 2021). In our study, the internal consistency coefficient was 0.93 for the MSS total score, 0.90, 0.72, and 0.91 for the positive, negative, and disorganized dimensions of the MSS, respectively.

The Autism‐Spectrum Quotient (AQ) (Baron‐Cohen et al. 2001) is a 50‐item self‐report scale for measuring autistic traits and has the social skills, attention switching, attention to detail, communication, and imagination subscales. The Chinese version of the AQ demonstrated high reliability and validity (Zhang et al. 2016). In our study, the internal consistency coefficient of the AQ total score was 0.66.

The Anticipatory and Consummatory Interpersonal Pleasure Scale (ACIPS) (Gooding and Pflum 2014) is a 17‐item self‐report scale for evaluating pleasure experiences during social interactions. Higher scores indicated more pleasure. The Chinese version of the ACIPS had high reliability and validity (Chan et al. 2016). In our study, the internal consistency coefficient of the ACIPS was 0.89.

The Berkley Expressivity Questionnaire (BEQ) (Gross and John 1995) is a 16‐item self‐report scale for measuring subjective expressivity. The BEQ has five subscales, namely positive expressivity, negative expressivity, negative inhibition, positive impulse strength, and negative impulse strength. The Chinese version of the BEQ had high validity and reliability (Zhao et al. 2015). In our study, the internal consistency coefficient of the BEQ was 0.83.

The Positive and Negative Affect Scale (PANAS) (Watson et al. 1988) measures participants' mood of the moment. It contains the positive affect and the negative affect subscales, each having 10 adjectives. The PANAS demonstrated good reliability and validity in a Chinese sample (Huang et al. 2003). In our study, the internal consistency coefficient of the PANAS was 0.83.

### 2.3 Data Analysis

Independent sample t‐tests were performed to examine sex differences on self‐report scales and the variables generated from computerized analysis for facial emotion expressions during film elicitation and autobiographical recalling tasks, that is, intensity of specific emotional expressions, valence, and arousal. Partial correlation analyses were conducted to examine the associations between schizotypal traits (MSS), autistic traits (AQ), social pleasure (ACIPS total score), and the variables from computerized analysis of facial expressions, after controlling for the sex variable. Principal component analysis (PCA) for the specific facial expressions across five conditions (neutral, negative, and positive videos watching; negative and positive autobiographical events recalling) was used to extract the score of the first principal component (PC) of each facial expression (percentages of variance explained: 54.13% for angry, 67.60% for surprised, 60.57% for scared, and 63.54% for arousal). We further examined the moderation effects of the PC for each facial expression on the associations between schizotypal/autistic traits and social pleasure. Moderation effect analysis was performed using Model 1 of Hayes's PROCESS, with sex as a covariate. Data analyses were conducted using SPSS v22. The significance threshold was set at *p* < 0.05.

## 3 Results

As shown in Table 1, male participants scored significantly higher on the AQ total score (*t*_(79)_ = 2.23, *p* = 0.029) and lower on the BEQ total score (*t*_(79)_ = −2.60, *p* = 0.011) than female participants. The two sex groups did not differ in the MSS and ACIPS.

**TABLE 1:** Description and sex differences on self‐report scales.

	Whole sample (*n* = 86)	Males (*n* = 42)	Females (*n* = 44)	Sex differences *t*_(df)_, *p*
Age (years)	20.35 (1.24)	20.69 (1.28)	20.02 (1.13)	
Length of education (years)	14.12 (1.02)	14.24 (1.03)	14.00 (1.01)	
MSS total	10.61 (9.03)	11.00 (11.03)	10.25 (6.77)	*t*_(83)_ = 0.38, *p* = 0.704
MSS positive	1.86 (3.62)	1.93 (4.63)	1.80 (2.37)	*t*_(83)_ = 0.17, *p* = 0.868
MSS negative	6.20 (3.60)	6.22 (3.53)	6.18 (3.71)	*t*_(83)_ = 0.05, *p* = 0.962
MSS disorganized	2.55 (4.25)	2.85 (4.93)	2.27 (3.53)	*t*_(83)_ = 0.63, *p* = 0.532
AQ total	20.32 (5.41)	21.71 (5.48)	19.09 (5.11)	*t*_(79)_ = 2.23, ***p* = 0.029**
AQ social skill	3.96 (2.21)	4.08 (2.44)	3.86 (2.01)	*t*_(79)_ = 0.44, *p* = 0.660
AQ attention switching	5.86 (1.72)	5.92 (1.75)	5.81 (1.72)	*t*_(79)_ = 0.28, *p* = 0.782
AQ attention to detail	4.73 (2.26)	5.21 (2.15)	4.30 (2.31)	*t*_(79)_ = 1.83, *p* = 0.071
AQ communication	2.73 (1.90)	3.11 (2.08)	2.40 (1.68)	*t*_(79)_ = 1.70, *p* = 0.093
AQ imagination	3.04 (1.39)	3.39 (1.33)	2.72 (1.39)	*t*_(79)_ = 2.23, ***p* = 0.029**
ACIPS total	77.93 (11.32)	78.74 (12.15)	77.17 (10.58)	*t*_(79)_ = 0.62, *p* = 0.534
ACIPS anticipatory	30.72 (4.38)	31.21 (4.69)	30.26 (4.08)	*t*_(79)_ = 0.97, *p* = 0.336
ACIPS consummatory	47.21 (7.33)	47.54 (7.77)	46.90 (6.98)	*t*_(79)_ = 0.39, *p* = 0.700
BEQ total	85.68 (9.78)	82.85 (10.63)	88.31 (8.20)	*t*_(79)_ = −2.60, ***p* = 0.011**
BEQ positive expressivity	16.35 (2.63)	15.95 (3.10)	16.71 (2.09)	*t*_(66)_ = −1.30, *p* = 0.200
BEQ negative expressivity	16.74 (4.07)	15.85 (4.52)	17.57 (3.45)	*t*_(79)_ = −1.94, *p* = 0.056
BEQ negative inhibition	13.86 (2.90)	14.44 (2.69)	13.33 (3.01)	*t*_(79)_ = 1.73, *p* = 0.087
BEQ positive impulse strength	14.54 (3.02)	14.21 (2.93)	14.86 (3.10)	*t*_(79)_ = −0.97, *p* = 0.335
BEQ negative impulse strength	14.05 (3.18)	12.85 (3.33)	15.17 (2.60)	*t*_(79)_ = −3.51, ***p* < 0.001**

*Note:* Bold indicates statistically significant values.

Abbreviations: ACIPS, The Anticipatory and Consummatory Interpersonal Pleasure Scale; AQ, The Autism‐Spectrum Quotient; BEQ, The Berkley Expressivity Questionnaire; MSS, Multidimensional Schizotypy Scale.

### 3.1 Descriptive Statistics on Computerized Analysis of Facial Expressions and Sex Effects

**FIGURE 2:**
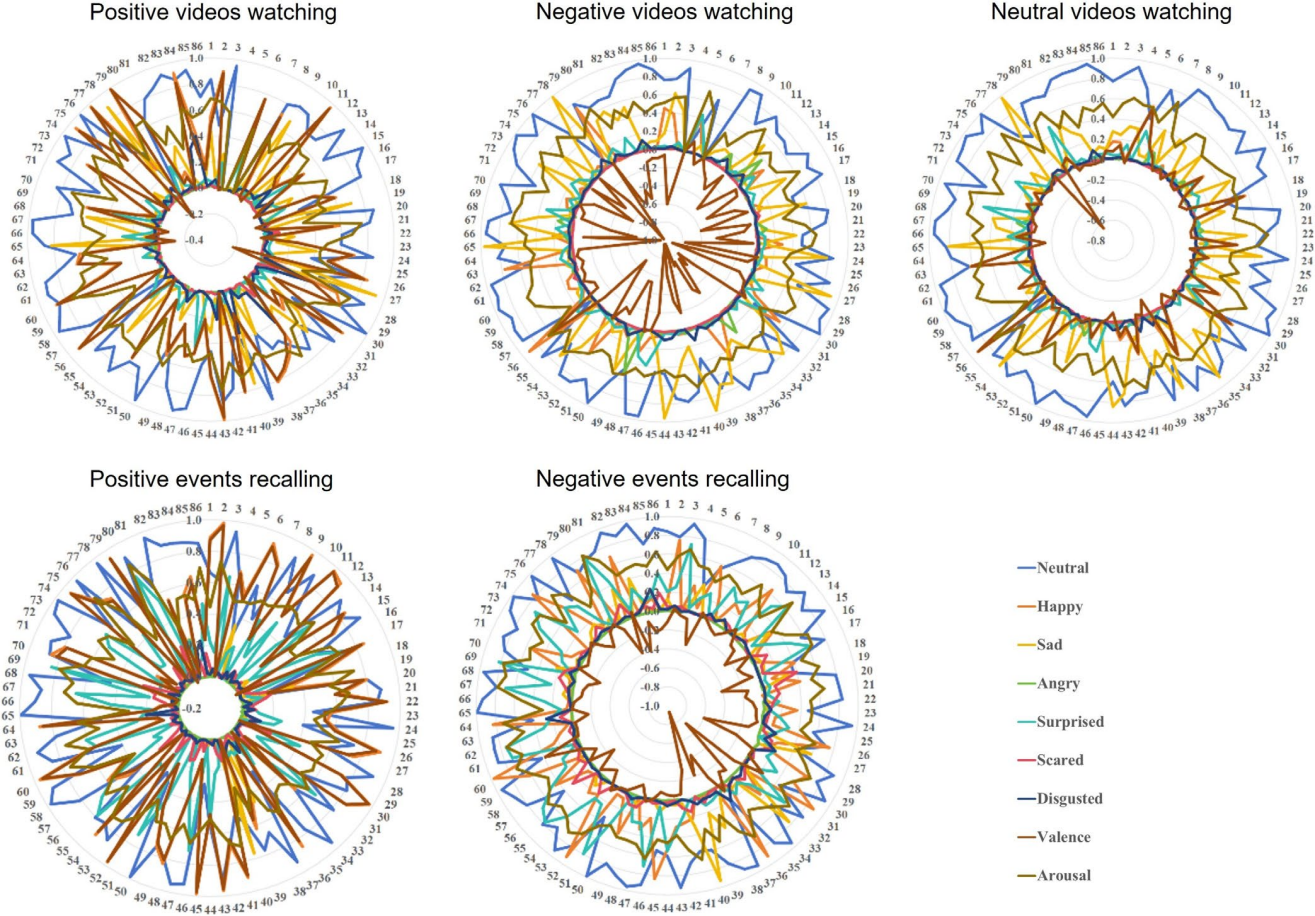
Individualized facial emotion expressions during film clips watching and autobiographical recalling tasks. Numbers of the outermost ring refer to each participant, numbers of the inner ring refer to intensity.

**FIGURE 3:**
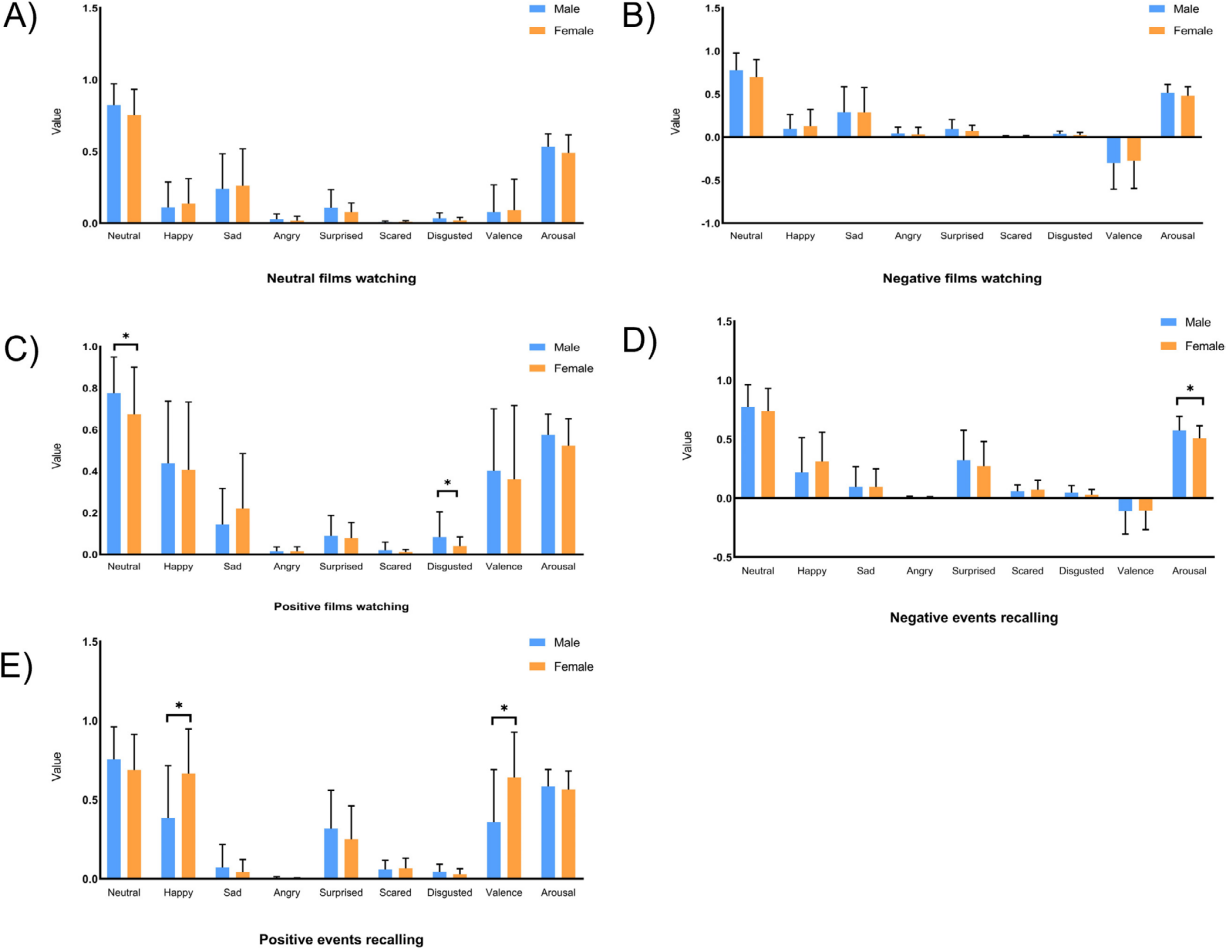
Descriptive statistics on computerized analysis of facial expressions and sex effects during film clip watching and autobiographical recalling tasks. (A) Specific indicators from FaceReader during neutral film watching (neutral, happy, sad, angry, surprised, scared, disgusted, valence, arousal); (B) Specific indicators from FaceReader during negative film watching; (C) Specific indicators from FaceReader during positive film watching; (D) Specific indicators from FaceReader during negative event recalling; (E) Specific indicators from FaceReader during positive event recalling; *p < 0.05.

Individualized data of facial expressions during film clips watching and autobiographical recalling is shown in Figure 2 and Table S1. Compared to female participants, male participants showed greater intensity in neutral (*t*_(81)_ = 2.25, *p* = 0.027) and disgusted (*t*_(47)_ = 2.09, *p* = 0.042) expressions during positive videos watching. For the autobiographical recalling task, male participants showed lower valence (*t*_(84)_ = −4.25, *p* < 0.001) and intensity of happy facial expression (*t*_(84)_ = −4.23, *p* < 0.001) when recalling positive events, and they also showed higher arousal during negative events recalling (*t*_(84)_ = 2.79, *p* = 0.006). Details are shown in Figure 3 and Table S2.

### 3.2 Associations Between Subclinical Traits and Social Pleasure

For schizotypal traits, we found negative correlations between the ACIPS total score and all dimensions of the schizotypal traits (positive: *r* = −0.26, *p* = 0.022; negative: *r* = −0.42, *p* < 0.001; disorganized: *r* = −0.30, *p* = 0.006). For autistic traits, we found negative relationships between the ACIPS total score and two AQ subscale scores, that is, social skills (*r* = −0.39, *p* < 0.001) and communication (*r* = −0.30, *p* = 0.007). These results indicated that higher levels of subclinical schizotypal or autistic traits were associated with less social pleasure.

### 3.3 Subclinical Traits and Facial Expressions During Film Elicitation Task

**FIGURE 4:**
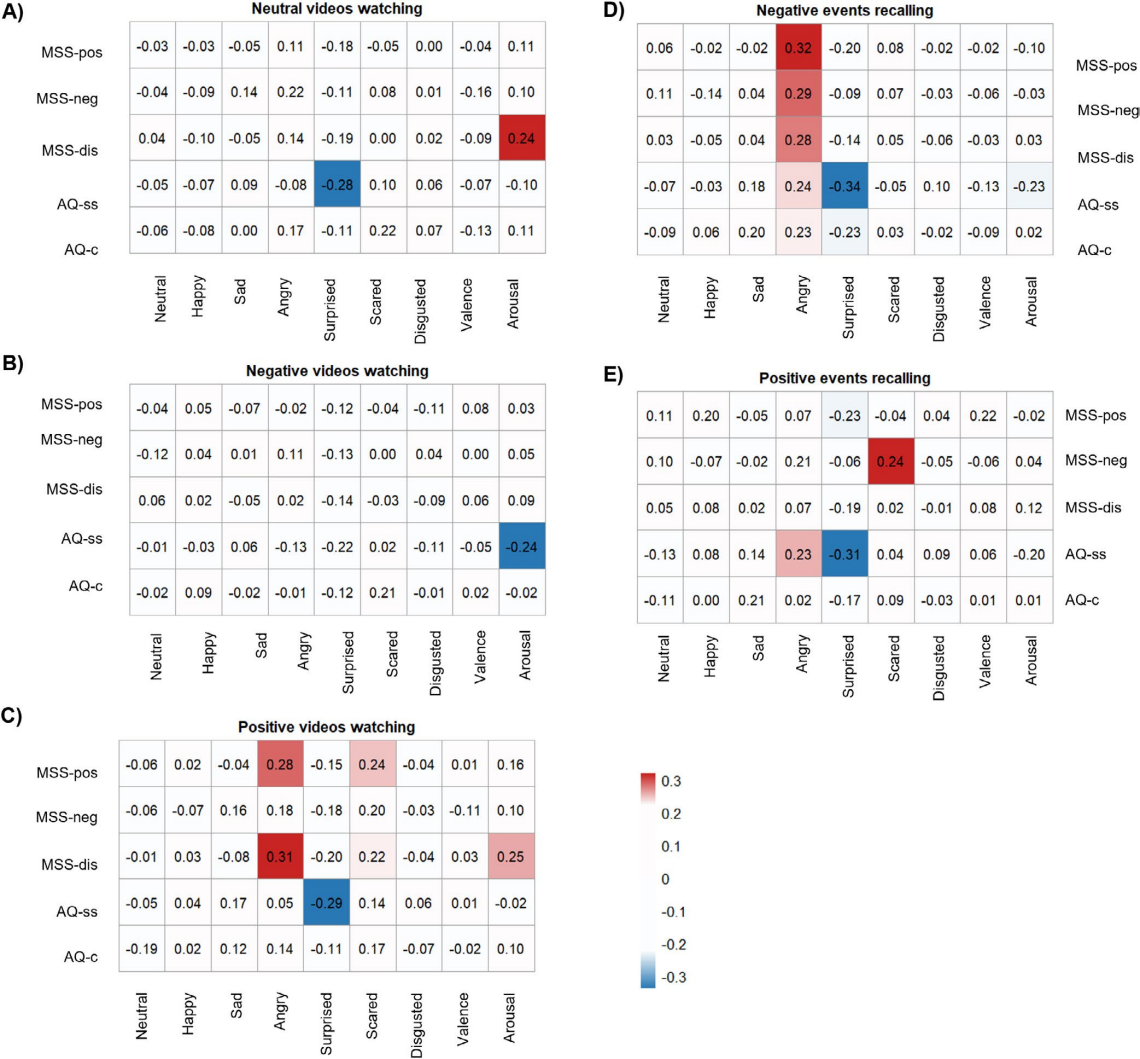
Partial correlations between MSS or AQ and computerized analysis of specific facial expressions under five different conditions, controlling for sex. AQ‐c = AQ communication, AQ‐ss = AQ social skill, MSS‐dis = MSS disorganized schizotypy, MSS‐neg = MSS negative schizotypy, MSS‐pos = MSS positive schizotypy. Panel (A–E): Partial correlations between MSS or AQ with specific indicators from FaceReader (A) during neutral videos watching, (B) during negative videos watching, (C) during positive videos watching, (D) during negative events recalling, (E) during positive events recalling.

As shown in Figure 4, the MSS positive dimension was positively correlated with the intensities of angry (*r* = 0.28, *p* = 0.012) and scared (*r* = 0.24, *p* = 0.029) facial expressions during positive videos watching; the MSS disorganized dimension was also positively correlated with the intensities of angry (*r* = 0.31, *p* = 0.005) and scared (*r* = 0.22, *p* = 0.045) facial expressions during positive videos watching. Lastly, the MSS disorganized dimension was positively correlated with the arousal during neutral (*r* = 0.24, *p* = 0.028) and positive (*r* = 0.25, *p* = 0.027) videos watching.

Regarding autistic trait, the AQ social skills subscale was negatively correlated with the intensity of surprised facial expression during neutral (*r* = −0.28, *p* = 0.014) and positive (*r* = −0.29, *p* = 0.011) video watching, and the arousal during negative videos watching (*r* = −0.24, *p* = 0.032).

### 3.4 Subclinical Traits and Facial Expressions During Autobiographical Recalling Task

All dimensions of the MSS showed positive correlations with the intensity of angry facial expression during negative events recalling (positive: *r* = 0.32, *p* = 0.004; negative: *r* = 0.29, *p* = 0.008; disorganized: *r* = 0.28, *p* = 0.011). During positive events recalling, the MSS positive dimension was negatively correlated with the intensity of surprised facial expression (*r* = −0.23, *p* = 0.036), while the MSS negative dimension was positively correlated with the intensity of scared facial expression (*r* = 0.24, *p* = 0.030).

The AQ social skills subscale was positively correlated with the intensity of angry facial expression when recalling positive (*r* = 0.23, *p* = 0.041) and negative events (*r* = 0.24, *p* = 0.036) but was negatively correlated with the intensity of surprised expression when recalling positive (*r* = −0.31, *p* = 0.005) and negative (*r* = −0.34, *p* = 0.002) events. In addition, the AQ social skills subscale was negatively correlated with arousal during negative events recalling (*r* = −0.23, *p* = 0.046). The AQ communication subscale was correlated with the intensity of angry (*r* = 0.23, *p* = 0.039) and surprised (*r* = −0.23, *p* = 0.043) facial expressions during negative events recalling. The results are shown in Figure 4 and detailed in Table S3.

### 3.5 Moderation Effect of Facial Expressivity on the Relationship Between Schizotypal Traits and Social Pleasure

**FIGURE 5:**
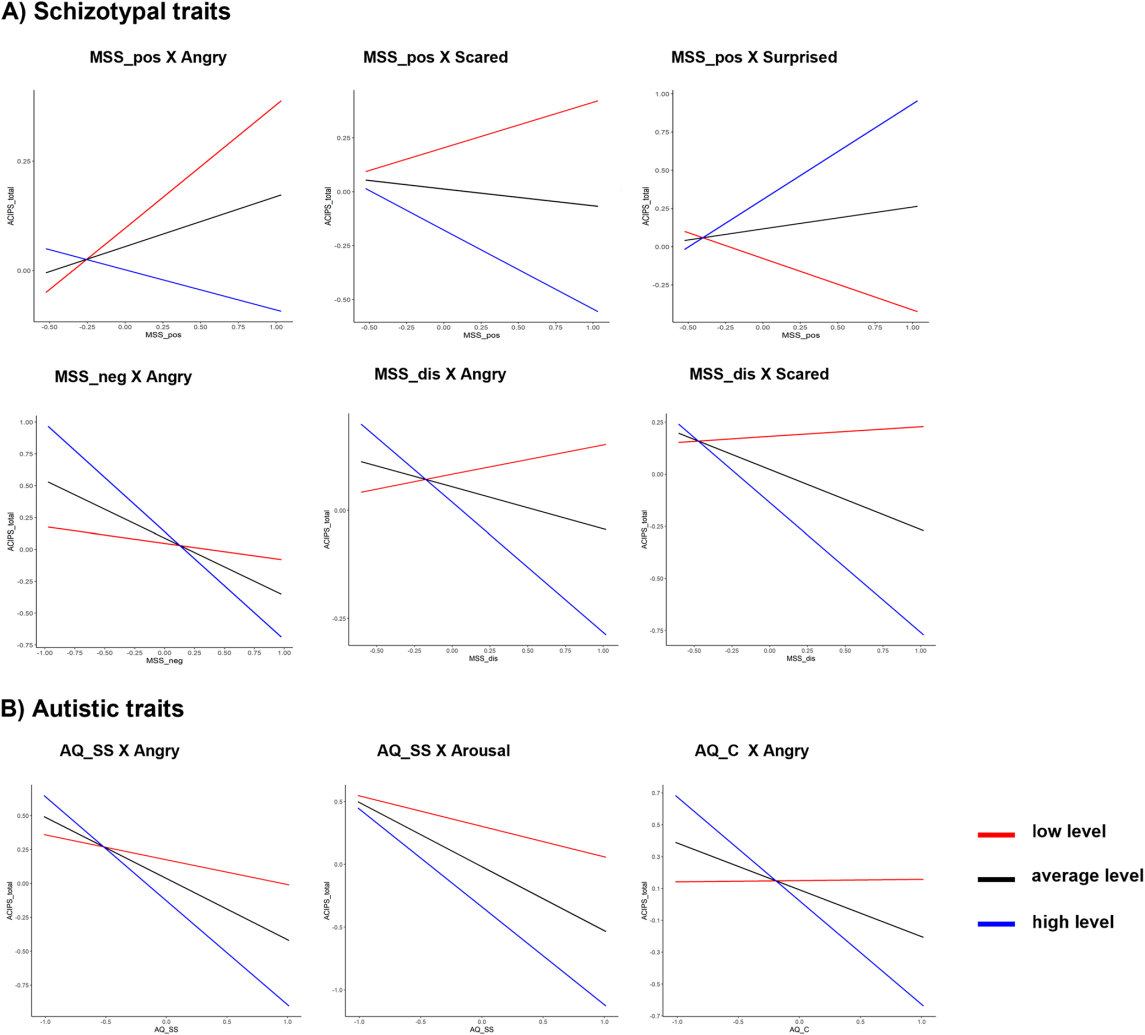
The relationship between MSS or AQ and ACIPS with emotion expressivity as the moderator. AQ‐c = AQ communication, AQ‐ss = AQ social skills, MSS‐dis = MSS disorganized schizotypy, MSS‐neg = MSS negative schizotypy, MSS‐pos = MSS positive schizotypy. Panel (A) shows moderating role of emotion expressivity in the relationship between MSS and ACIPS; Panel (B) shows moderating role of emotion expressivity in the relationship between AQ and ACIPS.

Table 2 and Figure 5 show the moderating effect of the facial expressions. The intensity of the angry facial expression had significantly moderated the negative relationships between the MSS (positive, negative, and disorganized) dimensions and the ACIPS (see Table 2 Model 1–3). Simple slope analysis showed that a higher MSS negative dimension was significantly associated with less social pleasure among individuals with an average or high (but not low) intensity of the angry facial expression. Moreover, a higher MSS disorganized dimension was significantly associated with less social pleasure in individuals with a high (but not average or low) intensity of the angry facial expression. However, the other models with the MSS positive dimension as the independent variable were non‐significant at any intensity level of the angry facial expression. Details are shown in Table S4.

**TABLE 2:** Moderating effect of facial emotion expressivity on the relationship between MSS and ACIPS.

		*β*	SE	*t*	LLCI	ULCI	*R* ^2^	∆*R* ^2^
Model 1	Constant	0.567	0.347	1.635	−0.124	1.258	0.220	0.132
	MSS positive	0.115	0.144	0.797	−0.172	0.401		
	Angry‐factor	−0.053	0.110	−0.482	−0.273	0.167		
	**MSS positive × Angry**	−0.208	0.059	−3.521***	−0.325	−0.09		
	Covariate: Sex	−0.333	0.213	−1.560	−0.758	0.092		
Model 2	Constant	0.568	0.322	1.762	−0.074	1.210	0.326	0.126
	MSS negative	−0.452	0.103	−4.392***	−0.656	−0.247		
	Angry‐factor	0.051	0.107	0.473	−0.163	0.264		
	**MSS negative × Angry**	−0.401	0.108	−3.697***	−0.617	−0.185		
	Covariate: Sex	−0.311	0.197	−1.578	−0.704	0.082		
Model 3	Constant	0.547	0.348	1.571	−0.147	1.241	0.210	0.098
	MSS disorganized	−0.097	0.124	−0.779	−0.344	0.150		
	Angry‐factor	−0.036	0.112	−0.323	−0.260	0.187		
	**MSS disorganized × Angry**	−0.206	0.068	−3.013**	−0.342	−0.07		
	Covariate: Sex	−0.320	0.214	−1.495	−0.747	0.107		
Model 4	Constant	0.362	0.347	1.044	−0.329	1.053	0.190	0.085
	MSS positive	−0.078	0.122	−0.639	−0.321	0.165		
	Scared‐factor	−0.187	0.105	−1.777	−0.396	0.023		
	**MSS positive × Scared**	−0.282	0.102	−2.774**	−0.485	−0.080		
	Covariate: Sex	−0.227	0.214	−1.058	−0.654	0.201		
Model 5	Constant	0.439	0.341	1.287	−0.241	1.118	0.224	0.097
	MSS disorganized	−0.288	0.104	−2.784**	−0.495	−0.082		
	Scared‐factor	−0.155	0.103	−1.510	−0.36	0.050		
	**MSS disorganized × Scared**	−0.328	0.109	−3.013**	−0.544	−0.111		
	Covariate: Sex	−0.270	0.211	−1.283	−0.690	0.150		
Model 6	Constant	0.706	0.397	1.780	−0.084	1.497	0.155	0.076
	MSS positive	0.144	0.186	0.772	−0.227	0.514		
	Surprised‐factor	0.191	0.123	1.555	−0.054	0.435		
	**MSS positive × Surprised**	0.473	0.184	2.569*	0.106	0.840		
	Covariate: Sex	−0.383	0.237	−1.616	−0.856	0.090		

Abbreviations: *β*, standardized regression coefficient; CI, confidence interval; LLCI, lower level of confidence interval; MSS, Multidimensional Schizotypy Scale; SE, standard error; ULCI, upper level of confidence interval.

*p < 0.05, **p < 0.01, ***p < 0.001.

The intensity of scared facial expression significantly moderated the negative relationships between the MSS positive and disorganized dimensions and the ACIPS (Table 2 Model 4–5). Simple slope analysis showed that the higher MSS positive dimension was significantly associated with less social pleasure only among individuals with high (but not average or low) intensity of scared expression, while the higher MSS disorganized dimension was significantly associated with less social pleasure among individuals with average or high (but not low) intensity of scared expression.

Lastly, the intensity of surprised facial expressions significantly moderated the negative relationship between the MSS positive dimension and the ACIPS (Table 2 Model 6). Simple slope analysis showed that a higher MSS positive dimension was significantly associated with less social pleasure in individuals with low (but not average or high) intensity of surprised expression.

### 3.6 Moderation Effect of Facial Emotion Expressivity on the Relationship Between Autistic Traits and Social Pleasure

The intensity of angry facial expression significantly moderated the negative relationship between the AQ social skills, the AQ communication, and the ACIPS (Table 3 Model 1–2). Simple slope analysis showed that higher AQ social skills and AQ communication were significantly associated with less social pleasure among individuals with average or high (but not low) intensity of angry expression. Arousal also significantly moderated the negative relationship between the AQ social skills and the ACIPS (Table 3 Model 3). Simple slope analysis showed that a higher AQ social skills subscale score was significantly associated with less social pleasure among individuals with average or high (but not low) levels of arousal. Details are shown in Table S5.

**TABLE 3:** Moderating effect of facial emotion expressivity on the relationship between AQ and ACIPS.

		*β*	SE	*t*	LLCI	ULCI	*R* ^2^	∆*R* ^2^
Model 1	Constant	0.424	0.337	1.258	−0.248	1.095	0.270	0.084
	AQ social skill	−0.451	0.104	−4.347***	−0.658	−0.244		
	Angry‐factor	−0.182	0.115	−1.588	−0.411	0.047		
	**AQ social skill × Angry**	−0.351	0.123	−2.850**	−0.597	−0.106		
	Covariate: Sex	−0.249	0.207	−1.207	−0.662	0.163		
Model 2	Constant	0.811	0.354	2.288*	0.104	1.518	0.242	0.128
	AQ communication	−0.294	0.107	−2.736**	−0.508	−0.08		
	Angry‐factor	−0.076	0.118	−0.644	−0.311	0.159		
	**AQ communication × Angry**	−0.396	0.114	−3.462**	−0.624	−0.168		
	Covariate: Sex	−0.463	0.217	−2.136**	−0.896	−0.031		
Model 3	Constant	0.604	0.347	1.739	−0.089	1.297	0.276	0.057
	AQ social skill	−0.511	0.108	−4.751***	−0.726	−0.297		
	Arousal‐factor	−0.318	0.112	−2.830**	−0.542	−0.094		
	**AQ social skill × Arousal**	−0.265	0.112	−2.367*	−0.489	−0.042		
	Covariate: Sex	−0.401	0.213	−1.882	−0.825	0.024		

Abbreviations: *β*, standardized regression coefficient; AQ, The Autism‐Spectrum Quotient; CI, confidence interval; LLCI, lower level of confidence interval; SE, standard error; ULCI, upper level of confidence interval.

*p < 0.05, **p < 0.01, ***p < 0.001.

## 4 Discussion

This study examined the relationship of schizotypal and autistic traits with social pleasure and explored the mechanisms of emotion expressivity underlying these associations. Our main findings can be summarized as follows. First, the three dimensions of schizotypal traits and the two dimensions of autistic traits were all negatively associated with social pleasure. Moreover, the intensity of angry facial expression moderated the relationship between schizotypal/autistic traits and social pleasure. Third, the intensity of surprised and scared facial expressions moderated the relationship between schizotypal traits and social pleasure. Lastly, the arousal level of facial expression was negatively correlated with autistic traits and moderated the relationship between autistic traits and social pleasure.

Our findings of the association between schizotypal traits and social pleasure aligned with previous results, which had shown that schizotypal traits were negatively correlated with social pleasure (Gooding et al. 2014) and positively correlated with social anhedonia (Chan et al. 2012). The use of the MSS had better captured the schizotypal dimension than previous similar studies (Gooding et al. 2014; Chan et al. 2012). In fact, a recent network analysis also found that the negative schizotypal dimension was negatively correlated with the anticipatory subscale of the ACIPS (Zhang et al. 2020). On the other hand, the AQ social skills and the AQ communication were negatively correlated with social pleasure, largely consistent with previous network analysis findings (Zhang et al. 2020). Clinical studies consistently demonstrated reduced social pleasure in both schizophrenia and ASD patients (Ritsner et al. 2018; Han et al. 2019). Together, our findings in subclinical samples corroborated the findings in clinical samples, that is, schizophrenia and ASD were associated with reduced social pleasure. Higher schizotypal traits corresponded with increased intensity of angry expression, particularly during positive videos watching and negative events recalling. People with higher schizotypal traits experienced less social pleasure if the intensity of angry expression reached the medium level. The existing evidence showed that both clinical (Tripoli et al. 2022) and subclinical (Statucka and Walder 2017) individuals of the schizophrenia spectrum recognized angry expressions less well than healthy people, and easily misinterpreted neutral facial expressions as angry (Premkumar et al. 2008; van Rijn et al. 2011). Moreover, schizophrenia patients reported more negative emotions in response to neutral and positive emotional stimuli (Cohen and Minor 2010; Riehle et al. 2023), and people with positive schizotypy were sensitive to angry voices (Papousek et al. 2014). Previous evidence also showed that schizophrenia patients expressed angry facial expressions less accurately than healthy people (Trémeau et al. 2005), and clinical high‐risk (CHR) individuals expressed angry facial expressions more frequently during face‐to‐face interviews (Gupta et al. 2019).

In addition, the higher the autistic traits, the greater the intensity of angry expressions during autobiographic recalling (regardless of valence). People with higher autistic traits experienced less pleasure in social interactions if the intensity of angry expression reached the medium level. The existing evidence showed that both children with autism and adults with high autistic traits recognized angry expressions less accurately than healthy people (Taylor et al. 2015; Macari et al. 2018; Poljac et al. 2013). Besides, ASD patients exhibited a higher intensity of angry expressions than healthy people (Faso et al. 2015).

Emotion perception, recognition, and experience were closely linked to emotional expression in social contexts (van Kleef and Côté 2022). The theory of “Emotion as Information in Early Social Learning” posits that people imitate emotions early in life as a form of social information. Abnormal recognition of a specific emotion type can lead to problems in expressing the particular emotion (Wu et al. 2021). Social interactions are dynamic, with an individual's expressions potentially affecting the emotional responses and social willingness of another individual. Angry expression conveys aggression and hostility (Chester and DeWall 2017), and may be perceived by others as signals of rejection, exclusion, or non‐cooperation (Heerdink et al. 2013; Van Doorn et al. 2012). Therefore, angry expression may elicit another individual's negative emotion (van Kleef and Côté 2022), and thus undermine social pleasure.

Higher positive schizotypal traits were associated with lower surprised expression intensity, particularly during positive events recalling. The existing evidence showed that schizophrenia patients had lower intensity and accuracy in surprised expressions than healthy people (Putnam and Kring 2007); and people with high schizotypy recognized surprised expressions less accurately than people with low schizotypy (Apolline et al. 2023). Unlike the negative and disorganized schizotypal traits, the positive schizotypal traits were specifically linked to lower recognition accuracy (Statucka and Walder 2017). Our findings concurred with the previous findings (Putnam and Kring 2007), suggesting a close relationship between positive schizotypal traits and the recognition and expression of surprised emotion.

The AQ social skills subscale was negatively associated with the intensity of surprised expression during watching neutral and positive videos, and during positive and negative events recalling. The existing evidence suggested that children with ASD recognized surprised expressions less accurately than healthy people (Baron‐Cohen et al. 1993; Balconi and Carrera 2007), and struggled to decide on surprised emotion. As such, the previous evidence and our findings appeared convergent.

In this study, the intensity of surprised expression moderated the relationship between schizotypal traits and social pleasure but did not moderate the relationship between autistic traits and social pleasure. Surprised expression may serve different roles in social interactions for people with schizotypal traits and people with autistic traits. The existing evidence suggested that the valence of surprised emotion varied with the context (Neta and Kim 2023), and therefore recognition and expression of surprised emotion could be influenced by social context and emotional learning. The intensity of scared expression was positively correlated with the three MSS dimensions, especially during positive film watching and events recalling. Previous evidence suggested that first‐episode schizophrenia patients recognized scared expressions less accurately than healthy people (Tripoli et al. 2022). In the general population, disorganized schizotypal traits and the accuracy of recognizing scared expression were negatively correlated (Statucka and Walder 2017). Moreover, schizophrenia patients showed lower accuracy in expressing scared emotion than healthy controls (Trémeau et al. 2005). Our findings were therefore consistent with previous findings. Additionally, a significant moderation effect was observed. When the intensity of scared expression was high, individuals with positive or disorganized schizotypal traits experienced less pleasure in social interactions. Higher positive schizotypal traits were linked to more hallucinations and referential delusions, leading to greater scared expression (Kemp et al. 2018). We speculated that scared expressions may be detected by others, further impacting social interactions and reducing social pleasure.

Furthermore, we found that higher scores on AQ social skills were associated with lower arousal levels, particularly during negative videos watching and negative events recalling. Evidence suggested that ASD patients exhibited lower skin conductance responses when recognizing emotional images compared to healthy controls (Hubert et al. 2009). Our findings indicated that higher social skills deficits in autistic traits correspond to lower levels of arousal, consistent with previous research. Moreover, we found that arousal moderated the relationship between the social skills dimension of autistic traits and social pleasure. When arousal reached medium levels, autistic traits were negatively correlated with social pleasure. We measured arousal using the FaceReader, based on action units (AU), with higher arousal indicating greater facial muscle activation in response to emotional stimuli. Stronger emotional responses, especially in negative contexts, might lead to more pronounced negative emotions during social interactions, potentially reducing social pleasure.

Our findings also revealed a negative correlation between arousal and the social skills dimension of autistic traits, and arousal moderated the relationship between the social skills dimension of autistic traits and social pleasure. In contrast, a positive correlation was found between arousal and the disorganized dimension of schizotypal traits, though the moderation effect was not significant. These findings suggest that arousal may play different roles in the relationships between these traits and social pleasure, indicating the need for further experimental research to verify these effects.

This study has several limitations. First, the emotional videos elicitation task and the autobiographical recalling task did not fully simulate natural social interactions. Second, participants' emotion regulation was not measured in the study and might have confounded the results. Third, although we recorded facial expressions and vocal information during autobiographical emotion tasks, only the data regarding facial expressions was analyzed using the FaceReader. Future research should integrate multidimensional data and integrate acoustic features and facial expressions to identify risk markers. Lastly, we did not include clinical patients with schizophrenia.

To conclude, higher schizotypal and autistic traits were associated with reduced social pleasure. Using an emotional videos elicitation task and an autobiographical recalling task in the general population, we found that schizotypal traits were linked to increased intensity of angry and scared facial expressions, and arousal, which further moderated the relationship between schizotypal traits and social pleasure. Autistic traits were associated with increased intensity of angry expression, with both the intensity of angry expression and arousal moderating the relationship between autistic traits and social pleasure. The intensity of angry expression seemed to have a similar mechanism under the association between both traits and social pleasure, while the intensity of scared expression and arousal may reflect specific changes associated with each trait. These findings provided valuable insights into the role of emotional expression characteristics in social interactions for people with schizotypal and autistic traits. The use of automated facial expression analysis software illustrated the application of computational technology to research on emotion processing in the schizophrenia and autism spectrum.

## Conflicts of Interest

The authors declare no conflicts of interest.

## Supporting information


**Data S1.** Supporting Information.
