# Supplementary material for: Moderation Effect of Emotional Expressivity on the Associations Between Schizotypal Traits, Autistic Traits and Social Pleasure
Source: Psych J. 2025 Feb 24;14(4):545–59. doi: 10.1002/pchj.70003 (PMC12318599; doi:10.1002/pchj.70003)
Supplement: Supplementary file 1 — Data S1. Supporting Information. [file PCHJ-14-545-s001.docx]

**Supplementary Materials**

**Table S1**

**Descriptive statistics on computerized analysis of facial expressions**

|  | Whole sample  (n=86) | Males  (n=42) | Females  (n=44) | Gender effect  *t*(df), *p* |
| --- | --- | --- | --- | --- |
| **Neutral videos watching** | | | | |
| Neutral | 0.788(0.168) | 0.824(0.149) | 0.754(0.180) | *t*_(84)_=1.96, *p*=0.053 |
| Happy | 0.124(0.175) | 0.112(0.176) | 0.136(0.176) | *t*_(84)_=-0.62, *p*=0.540 |
| Sad | 0.251(0.250) | 0.240(0.244) | 0.261(0.258) | *t*_(84)_ =-0.38, *p*=0.707 |
| Angry | 0.023(0.034) | 0.027(0.037) | 0.019(0.030) | *t*_(84)_ =1.10, *p*=0.273 |
| Surprised | 0.094(0.098) | 0.109(0.125) | 0.080(0.061) | *t*_(59)_ =1.39, *p*=0.171 |
| Scared | 0.009(0.008) | 0.008(0.008) | 0.010(0.008) | *t*_(84)_=-0.62, *p*=0.537 |
| Disgusted | 0.028(0.031) | 0.035(0.039) | 0.021(0.021) | *t*_(62)_=2.13, *p*=0.037 |
| Valence | 0.086(0.202) | 0.079(0.189) | 0.093(0.215) | *t*_(84)_=-0.31, *p*=0.761 |
| Arousal | 0.512(0.111) | 0.534(0.089) | 0.491(0.125) | *t*_(78)_=1.84, *p*=0.070 |
| **Negative videos watching** | | | | |
| Neutral | 0.734(0.206) | 0.775(0.202) | 0.697(0.206) | *t*_(82)_=1.77, *p*=0.081 |
| Happy | 0.113(0.182) | 0.095(0.167) | 0.129(0.195) | *t*_(82)_=-0.85, *p*=0.401 |
| Sad | 0.290(0.290) | 0.291(0.295) | 0.290(0.288) | *t*_(82)_=0.01, *p*=0.990 |
| Angry | 0.037(0.078) | 0.042(0.075) | 0.033(0.082) | *t*_(82)_=0.51, *p*=0.612 |
| Surprised | 0.082(0.092) | 0.093(0.113) | 0.071(0.069) | *t*_(63)_=1.07, *p*=0.290 |
| Scared | 0.009(0.011) | 0.009(0.011) | 0.010(0.012) | *t*_(82)_=-0.31, *p*=0.756 |
| Disgusted | 0.032(0.032) | 0.038(0.034) | 0.026(0.030) | *t*_(82)_=1.69, *p*=0.095 |
| Valence | -0.288(0.310) | -0.304(0.0.303) | -0.276(0.320) | *t*_(82)_=-0.38, *p*=0.703 |
| Arousal | 0.497(0.103) | 0.515(0.098) | 0.481(0.106) | *t*_(82)_=1.52, *p*=0.133 |
| **Positive videos watching** | | | | |
| Neutral | 0.721(0.209) | 0.775(0.175) | 0.674(0.227) | ***t*_(81)_=2.25, *p*=0.027** |
| Happy | 0.421(0.312) | 0.438(0.299) | 0.406(0.327) | *t*_(81)_=0.46, *p*=0.646 |
| Sad | 0.185(0.228) | 0.144(0.173) | 0.221(0.264) | *t*_(75)_=-1.58, *p*=0.118 |
| Angry | 0.016(0.021) | 0.016(0.020) | 0.016(0.021) | *t*_(81)_=0.04, *p*=0.965 |
| Surprised | 0.083(0.087) | 0.089(0.099) | 0.078(0.076) | *t*_(81)_=0.56, *p*=0.579 |
| Scared | 0.015(0.028) | 0.020(0.039) | 0.011(0.013) | *t*_(46)_=1.40, *p*=0.169 |
| Disgusted | 0.061(0.091) | 0.084(0.121) | 0.041(0.044) | ***t*_(47)_=2.09, *p*=0.042** |
| Valence | 0.380(0.328) | 0.402(0.298) | 0.361(0.355) | *t*_(81)_=0.56, *p*=0.574 |
| Arousal | 0.547(0.119) | 0.575(0.100) | 0.523(0.130) | *t*_(79)_=2.08, *p*=0.041 |
| **Negative events** **recalling** | | | | |
| Neutral | 0.755(0.191) | 0.774(0.190) | 0.737(0.193) | *t*_(84)_=0.88, *p*=0.384 |
| Happy | 0.265(0.275) | 0.218(0.295) | 0.310(0.250) | *t*_(84)_=-1.57, *p*=0.121 |
| Sad | 0.096(0.161) | 0.096(0.171) | 0.096(0.152) | *t*_(84)_=0.00, *p*=0.998 |
| Angry | 0.006(0.009) | 0.006(0.010) | 0.006(0.007) | *t*_(84)_=-0.10, *p*=0.924 |
| Surprised | 0.297(0.232) | 0.324(0.254) | 0.272(0.209) | *t*_(84)_=1.04, *p*=0.302 |
| Scared | 0.066(0.067) | 0.059(0.054) | 0.074(0.078) | *t*_(84)_=-1.03, *p*=0.308 |
| Disgusted | 0.040(0.052) | 0.049(0.059) | 0.031(0.044) | *t*_(84)_=1.60, *p*=0.112 |
| Valence | -0.107(0.177) | -0.109(0.194) | -0.104(0.162) | *t*_(84)_=-0.14, *p*=0.886 |
| Arousal | 0.540(0.118) | 0.575(0.120) | 0.507(0.107) | ***t*_(84)_=2.79, *p*=0.006** |
| **Positive events recalling** | | | | |
| Neutral | 0.722(0.217) | 0.756(0.205) | 0.689(0.225) | *t*_(84)_=1.43, *p*=0.156 |
| Happy | 0.529(0.336) | 0.385(0.330) | 0.665(0.283) | ***t*_(84)_=-4.23, *p<*0.000** |
| Sad | 0.057(0.117) | 0.072(0.146) | 0.043(0.080) | *t*_(63)_ =1.13, *p*=0.260 |
| Angry | 0.004(0.007) | 0.005(0.010) | 0.003(0.003) | *t*_(49)_=1.21, *p*=0.232 |
| Surprised | 0.283(0.228) | 0.318(0.242) | 0.250(0.212) | *t*_(84)_=1.39, *p*=0.167 |
| Scared | 0.063(0.061) | 0.060(0.058) | 0.066(0.064) | *t*_(84)_=-0.50, *p*=0.621 |
| Disgusted | 0.037(0.042) | 0.045(0.048) | 0.030(0.034) | *t*_(84)_=1.66, *p*=0.100 |
| Valence | 0.504(0.339) | 0.359(0.332) | 0.642(0.286) | ***t*_(84)_=-4.25, *p<*0.000** |
| Arousal | 0.574(0.112) | 0.584(0.107) | 0.565(0.117) | *t*_(84)_=0.79, *p*=0.430 |

**Table S2**

**Partial correlations between MSS/AQ and computerized analysis of specific facial expressions, controlling for sex**

|  | MSS-pos | MSS-neg | MSS-dis | AQ-ss | AQ-c |
| --- | --- | --- | --- | --- | --- |
| **Neutral videos watching** | | | | | |
| Neutral | -0.03 | -0.04 | 0.04 | -0.05 | -0.06 |
| Happy | -0.03 | -0.09 | -0.10 | -0.07 | -0.08 |
| Sad | -0.05 | 0.14 | -0.05 | 0.09 | 0.00 |
| Angry | 0.11 | 0.22 | 0.14 | -0.08 | 0.17 |
| Surprised | -0.18 | -0.11 | -0.19 | -0.28** | -0.11 |
| Scared | -0.05 | 0.08 | 0.00 | 0.10 | 0.22 |
| Disgusted | 0.00 | 0.01 | 0.02 | 0.06 | 0.07 |
| Valence | -0.04 | -0.16 | -0.09 | -0.07 | -0.13 |
| Arousal | 0.11 | 0.10 | 0.24* | -0.10 | 0.11 |
| **Negative videos watching** | | | | | |
| Neutral | -0.04 | -0.12 | 0.06 | -0.01 | -0.02 |
| Happy | 0.05 | 0.04 | 0.02 | -0.03 | 0.09 |
| Sad | -0.07 | 0.01 | -0.05 | 0.06 | -0.02 |
| Angry | -0.02 | 0.11 | 0.02 | -0.13 | -0.01 |
| Surprised | -0.12 | -0.13 | -0.14 | -0.22 | -0.12 |
| Scared | -0.04 | 0.00 | -0.03 | 0.02 | 0.21 |
| Disgusted | -0.11 | 0.04 | -0.09 | -0.11 | -0.01 |
| Valence | 0.08 | 0.00 | 0.06 | -0.05 | 0.02 |
| Arousal | 0.03 | 0.05 | 0.09 | -0.24* | -0.02 |
| **Positive videos watching** | | | | | |
| Neutral | -0.06 | -0.06 | -0.01 | -0.05 | -0.19 |
| Happy | 0.02 | -0.07 | 0.03 | 0.04 | 0.02 |
| Sad | -0.04 | 0.16 | -0.08 | 0.17 | 0.12 |
| Angry | 0.28* | 0.18 | 0.31** | 0.05 | 0.14 |
| Surprised | -0.15 | -0.18 | -0.20 | -0.29** | -0.11 |
| Scared | 0.24* | 0.20 | 0.22* | 0.14 | 0.17 |
| Disgusted | -0.04 | -0.03 | -0.04 | 0.06 | -0.07 |
| Valence | 0.01 | -0.11 | 0.03 | 0.01 | -0.02 |
| Arousal | 0.16 | 0.10 | 0.25* | -0.02 | 0.10 |
| **Negative events recalling** | | | | | |
| Neutral | 0.06 | 0.11 | 0.03 | -0.07 | -0.09 |
| Happy | -0.02 | -0.14 | -0.05 | -0.03 | 0.06 |
| Sad | -0.02 | 0.04 | 0.04 | 0.18 | 0.20 |
| Angry | 0.32** | 0.29** | 0.28* | 0.24* | 0.23* |
| Surprised | -0.20 | -0.09 | -0.14 | -0.34* | -0.23* |
| Scared | 0.08 | 0.07 | 0.05 | -0.05 | 0.03 |
| Disgusted | -0.02 | -0.03 | -0.06 | 0.10 | -0.02 |
| Valence | -0.02 | -0.06 | -0.03 | -0.13 | -0.09 |
| Arousal | -0.10 | -0.03 | 0.03 | -0.23* | 0.02 |
| **Positive events recalling** | | | | | |
| Neutral | 0.11 | 0.10 | 0.05 | -0.13 | -0.11 |
| Happy | 0.20 | -0.07 | 0.08 | 0.08 | 0.00 |
| Sad | -0.05 | -0.02 | 0.02 | 0.14 | 0.21 |
| Angry | 0.07 | 0.21 | 0.07 | 0.23* | 0.02 |
| Surprised | -0.23* | -0.06 | -0.19 | -0.31** | -0.17 |
| Scared | -0.04 | 0.24* | 0.02 | 0.04 | 0.09 |
| Disgusted | 0.04 | -0.05 | -0.01 | 0.09 | -0.03 |
| Valence | 0.22 | -0.06 | 0.08 | 0.06 | 0.01 |
| Arousal | -0.02 | 0.04 | 0.12 | -0.20 | 0.01 |

*Note.* MSS-pos= MSS positive schizotypy; MSS-neg= MSS negative schizotypy; MSS-dis= MSS disorganized schizotypy; AQ-ss: AQ social skill; AQ-c: AQ communication. **p < 0.05，**p < 0.01.*

**Table S3**

**Factor Loadings of the principal component analysis**

|  | **Angry** | **Surprised** | **Scared** | **Arousal** |
| --- | --- | --- | --- | --- |
| Neutral videos watching | 0.76 | 0.79 | 0.67 | 0.77 |
| Negative videos watching | 0.50 | 0.56 | 0.65 | 0.71 |
| Positive videos watching | 0.53 | 0.71 | 0.34 | 0.62 |
| Negative events recalling | 0.52 | 0.63 | 0.72 | 0.53 |
| Positive events recalling | 0.39 | 0.70 | 0.65 | 0.55 |
| **% Variance explained** | 54.13% | 67.60% | 60.57% | 63.54% |

**Table S4**

**Simple slopes for moderation effect analysis on the relationship between MSS and ACIPS**

| **Relation between MSS-pos and ACIPS** | |  |  |  |  |  |
| --- | --- | --- | --- | --- | --- | --- |
| **Angry** | **Effect** | ***SE*** | ***t*** | ***p*** | ***LLCI*** | ***ULCI*** |
| -0.801 | 0.281 | 0.180 | 1.566 | 0.122 | -0.077 | 0.639 |
| 0.000 | 0.115 | 0.144 | 0.797 | 0.428 | -0.172 | 0.401 |
| 0.992 | -0.092 | 0.112 | -0.819 | 0.415 | -0.315 | 0.131 |
| **Surprised** | **Effect** | ***SE*** | ***t*** | ***p*** | ***LLCI*** | ***ULCI*** |
| -1.015 | -0.337 | 0.115 | -2.927 | 0.005 | -0.566 | -0.107 |
| 0.000 | 0.144 | 0.186 | 0.772 | 0.443 | -0.227 | 0.514 |
| 1.015 | 0.624 | 0.355 | 1.759 | 0.083 | -0.083 | 1.331 |
| **Scared** | **Effect** | ***SE*** | ***t*** | ***p*** | ***LLCI*** | ***ULCI*** |
| -1.022 | 0.211 | 0.198 | 1.066 | 0.290 | -0.183 | 0.604 |
| 0.000 | -0.078 | 0.122 | -0.639 | 0.525 | -0.321 | 0.165 |
| 1.022 | -0.367 | 0.111 | -3.290 | 0.002 | -0.589 | -0.145 |
| **Relation between MSS-neg and ACIPS** | | | | | | |
| **Angry** | **Effect** | ***SE*** | ***t*** | ***p*** | ***LLCI*** | ***ULCI*** |
| -0.801 | -0.410 | 0.413 | -0.992 | 0.324 | -1.234 | 0.414 |
| 0.000 | -1.419 | 0.323 | -4.392 | 0.000 | -2.063 | -0.775 |
| 0.992 | -2.667 | 0.478 | -5.583 | 0.000 | -3.620 | -1.715 |
| **Relation between MSS-dis and ACIPS** | | | | | | |
| **Angry** | **Effect** | ***SE*** | ***t*** | ***p*** | ***LLCI*** | ***ULCI*** |
| -0.801 | 0.069 | 0.159 | 0.431 | 0.668 | -0.248 | 0.385 |
| 0.000 | -0.097 | 0.124 | -0.779 | 0.438 | -0.344 | 0.150 |
| 0.992 | -0.301 | 0.107 | -2.812 | 0.006 | -0.514 | -0.088 |
| **Scared** | **Effect** | ***SE*** | ***t*** | ***p*** | ***LLCI*** | ***ULCI*** |
| -1.022 | 0.046 | 0.155 | 0.300 | 0.765 | -0.263 | 0.356 |
| 0.000 | -0.288 | 0.104 | -2.784 | 0.007 | -0.495 | -0.082 |
| 1.022 | -0.623 | 0.149 | -4.193 | 0.000 | -0.920 | -0.327 |

*Note.* *SE*: standard error; *LLCI*: lower level of confidence interval; *ULCI*: upper level of confidence interval. MSS-pos= MSS positive schizotypy; MSS-neg= MSS negative schizotypy; MSS-dis= MSS disorganized schizotypy.

**Table S5**

Moderation model effect on the relationship between AQ and ACIPS upon different degrees (-1SD to 1SD)

| **Relation between AQ-ss and ACIPS** | | | | | | |
| --- | --- | --- | --- | --- | --- | --- |
| **Angry** | **Effect** | ***SE*** | ***t*** | ***p*** | ***LLCI*** | ***ULCI*** |
| -0.762 | -0.184 | 0.126 | -1.456 | 0.150 | -0.435 | 0.068 |
| 0.000 | -0.451 | 0.104 | -4.347 | 0.000 | -0.658 | -0.244 |
| 0.900 | -0.767 | 0.166 | -4.633 | 0.000 | -1.098 | -0.437 |
| **Arousal** | **Effect** | ***SE*** | ***t*** | ***p*** | ***LLCI*** | ***ULCI*** |
| -1.010 | -0.243 | 0.136 | -1.790 | 0.078 | -0.514 | 0.028 |
| 0.000 | -0.511 | 0.108 | -4.751 | 0.000 | -0.726 | -0.297 |
| 1.010 | -0.779 | 0.174 | -4.474 | 0.000 | -1.126 | -0.432 |
| **Relation between AQ-c and ACIPS** | | | | | | |
| **Angry** | **Effect** | ***SE*** | ***t*** | ***p*** | ***LLCI*** | ***ULCI*** |
| -0.762 | 0.008 | 0.139 | 0.057 | 0.954 | -0.270 | 0.285 |
| 0.000 | -0.294 | 0.107 | -2.736 | 0.008 | -0.508 | -0.080 |
| 0.900 | -0.651 | 0.148 | -4.399 | 0.000 | -0.945 | -0.356 |

*Note.* *SE*: standard error; *LLCI*: lower level of confidence interval; *ULCI*: upper level of confidence interval; AQ-ss: AQ social skill; AQ-c: AQ communication.

**Comparisons of self-report valence and arousal during emotion elicitation task.**

We performed repeated ANOVA and post hoc ANOVA on self-report valence and arousal. Results indicated that participants reported the highest valence during positive videos watching (*p*<0.001), followed by neutral videos, and then negative videos (*p*<0.001). Arousal during both negative and positive videos watching were reported higher than neutral videos (*p*<0.001). Details are presented in Table S6.

**Table S6**

**Descriptive statistics on self-report (valence, arousal)**

|  | **Neu films** | **Neg films** | **Pos films** | ***F*** (2, 85) | ***P*** |
| --- | --- | --- | --- | --- | --- |
| **Valence** | 4.895(0.058) | 3.308(0.100) | 6.750(0.117) | 313.075 | <0.001 |
| **Arousal** | 2.331(0.126) | 5.791(0.159) | 5.797(0.181) | 267.453 | <0.001 |

**Comparisons of PANAS score at three time points.**

We performed repeated ANOVA and post hoc ANOVA on positive and negative PANAS emotion scores. Participants were instructed to complete PANAS scale at three time points (Before tasks, After negative tasks, After positive tasks). Results indicated that participants experienced the highest negative emotion after negative tasks (*p*<0.001) and the highest positive emotion at T3 (after positive tasks) (*p*<0.001). Details are presented in Table S7.

**Table S7**

**Descriptive statistics on PANAS (The Positive and Negative Affect Scale)**

|  | **Before tasks** | **After negative tasks** | **After positive tasks** | ***F*** (2, 85) | ***P*** |
| --- | --- | --- | --- | --- | --- |
| **Positive emotion** | 28.640(0.814) | 25.349(0.817) | 32.174(0.858) | 54.791 | <0.001 |
| **Negative emotion** | 15.930(0.627) | 18.884(0.640) | 13.337(0.452) | 42.310 | <0.001 |
